# To close, not to close, or to act bigger? Managing the defect of large direct inguinal hernia to reduce the risk of recurrence during laparoscopic TAPP repair: a retrospective cohort study

**DOI:** 10.1007/s13304-024-01870-y

**Published:** 2024-05-11

**Authors:** F. Brucchi, F. Ferraina, E. Masci, D. Ferrara, D. Cassini, G. Faillace

**Affiliations:** 1 University of Milano Statale, via Festa del Perdono, 7, 20122 Milan, MI Italy; 2 ASST Nord Milano- Department of General Surgery, Sesto San Giovanni Hospital, Sesto San Giovanni, MI Italy; 3 ASST Nord Milano Department of General Surgery, Edoardo Bassini Hospital, Cinisello Balsamo, MI Italy; 4 University of Milano Bicocca, Piazza dell’Ateneo Nuovo, 1, 20126 Milan, Italy

**Keywords:** Inguinal hernia, Laparoscopy, TAPP, Direct inguinal hernia, Groin hernia surgery

## Abstract

Hernia recurrence is a common complication after inguinal hernia repair. Recent studies suggest that laparoscopic mesh repair with closure of direct hernia defects can reduce recurrence rates. Our study examines the effectiveness of this approach. A retrospective, multi-center cohort study was conducted on cases performed from January 2013 to April 2021. Patients with direct inguinal hernias (M3 according to EHS classification) undergoing TAPP were included. Three groups were present: closed-defect group, non-closed placing a standard-sized mesh group or non-closed placing an XL-sized mesh group. A 2-year follow-up was recorded. A total of 158 direct M3 inguinal hernias in 110 patients who underwent surgery were present. After propensity score matching at a 1:1 ratio, 22 patients for each group were analyzed. The mean age of patients was 62 years (41–84); with the majority being male (84.8%). 22 patients (40 hernias) underwent closure of the defect; 22 patients (39 hernias) did not undergo closure and used a standard-sized mesh; 22 patients (27 hernias) did not undergo closure and used an XL-sized mesh. There were 5 recurrences at 1 year post-operatively: all in the non-closure group with standard-sized mesh. This difference was statistically significant (*p* = 0.044). There were 7 recurrences (6.6%) at 2 years post-operatively: 6 in the non-closure group with standard-sized mesh and 1 in the non-closure group with XL-sized mesh (*p* = 0.007). Closing large direct inguinal hernia defects has shown promise in reducing early recurrence rates. However, conducting larger RCTs in the future could provide more conclusive evidence that might impact the way we approach laparoscopic inguinal hernia repair.

## Introduction

Laparoscopic surgery for hernia repair offers numerous advantages when compared to traditional open surgery, including a similar recurrence rate, a reduced risk of complications related to surgical wounds, quicker resumption of work and daily activities, and diminished likelihood of experiencing chronic pain [1]. Laparoscopic repair is now the recommended choice for bilateral or recurrent hernias after open repair. For primary unilateral inguinal hernias, laparoscopic repair is advisable if the surgeon is experienced in the procedure [2].

The risk of recurrence has significantly decreased over the last century due to technical advancements [3]. The current risk of recurrence after repair is less than 2%, and this appears to be higher in medial hernias compared to lateral ones [4]. However, recurrence remains a critical post-operative complication, impacting the quality of life and potentially leading to pain, incarceration, and the need for reoperation [5].

Among the risk factors to consider for recurrence, the size of the prosthesis is undoubtedly important. The available literature on the appropriate prosthesis size and overlap relative to the defect's dimensions is indeed quite limited. It suggests maintaining a minimum overlap of 3 cm when the defect exceeds 3 cm in diameter, necessitating the use of a larger prosthesis accordingly [6, 7].

It is essential to draw from the experience of defect closure in ventral hernias, which appears to be linked to a reduced risk of seroma formation and potential recurrences [8, 9]. The hypothesis is that this concept can be applied with similar outcomes to direct inguinal hernias, especially those with larger defects. However, there is a limited amount of available literature on this topic.

In the case of direct inguinal hernias, it is not a standard practice to close the defect during laparoscopic procedures, unlike in open repairs where the defect is typically sutured before placing the mesh. Consequently, there may be a heightened risk of mesh eventration, recurrence, and seroma formation following laparoscopic repair due to the absence of defect closure [10].

This study aimed to assess whether laparoscopic primary closure of large direct inguinal hernia defects with barbed suture or the positioning of a larger mesh is linked to a reduced risk of early recurrence and the formation of seromas.

## Materials and methods

### Patients

**Fig. 1:**
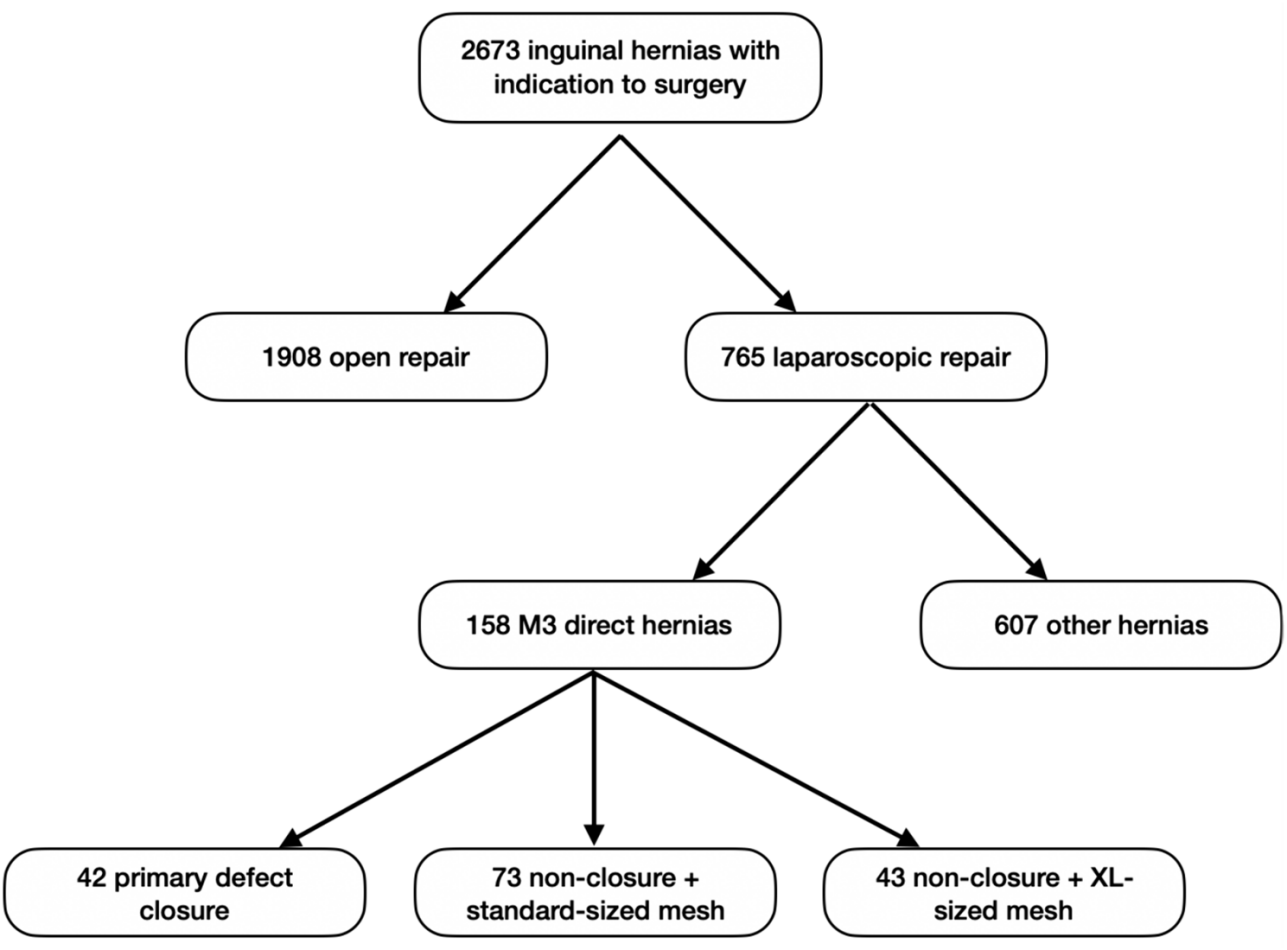
Flowchart showing the management of the patients in our department

We conducted a retrospective, multi-center cohort study covering surgeries performed between January 2013 and April 2021 at the Department of General Surgery, ASST-Nord Milano in Sesto San Giovanni City Hospital and in the E. Bassini Hospital of Cinisello Balsamo. The study focused on patients with large direct inguinal hernias, specifically those classified as M3 according to the EHS classification (Fig. 1) [11], who underwent elective laparoscopic repair. All other types of hernias were excluded from the study. All procedures were either conducted by the same surgeon or under their supervision. These surgeons, experts in TAPP hernia repair from a shared background, have collaboratively standardized their technique after each successfully completing over 700 procedures. They have decided to exclusively handle M3 hernia cases to streamline the approach, minimize biases arising from diverse techniques, and effectively address the challenge of recurrences in the study.

The study received approval from the hospital's ethics committee, and a waiver of consent was granted for this retrospective review. In accordance with the Helsinki Declaration, the study protocol has been registered on the researchregistry.com platform with the unique identifying number researchregistry9693 (https://www.researchregistry.com/browse-the-registry#home/registrationdetails/655279dc5ca7f6002758d067/).

### Surgical procedure

In our center, we predominantly use the Transabdominal Preperitoneal (TAPP) approach as the default surgical technique for laparoscopic hernia repair. This approach adheres to established and standardized methods outlined in the medical literature. All surgeries were performed under general anesthesia following intravenous antibiotic prophylaxis with 2 g of cefazolin.

**Fig. 2:**
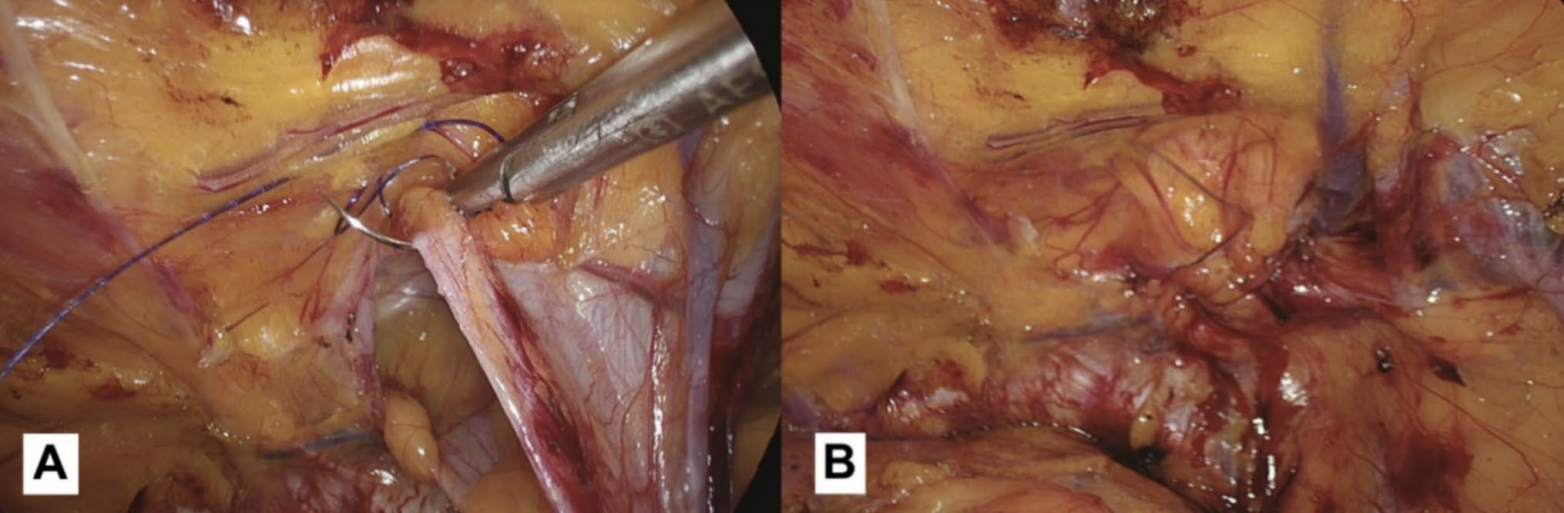
**A** In the case of M3 direct inguinal hernias, we recommend suturing the defect while paying attention to the elements of the spermatic cord. **B** The result obtained after the closure

The measurement of direct hernia defect size is conducted using the instrument tip length of the laparoscopic grasper as a reference gauge. In instances where closure of the direct defect is indicated, the hernia sac is initially repositioned into the preperitoneal space. Following this, closure of the transversalis fascia is carried out on both sides of the defect using a barbed suture with a synthetic absorbable monofilament (Polydioxanone) 2/0 Filbloc (Assut Europe). During this suture, ensuring a tension-free approach is essential. It is necessary to focus on the pseudosac during closure and avoid muscle involvement to keep the suture tension-free, consequently reducing the risk of chronic pain. Furthermore, it is crucial to ensure that the needle is inserted superficially, primarily through the upper part of the defect. This practice helps prevent any harm to the nearby spermatic cord structures and the iliohypogastric nerve (Fig. 2) [12].

A standard 10.3 cm × 15.7 cm polypropylene, large pore, 3D mesh (3DMaxTM Mesh, BD, 100 Crossings BoulevardWarwick, Rhode Island 02886, United States): a curved, pre-shaped, three-dimensional polypropylene prosthesis is subsequently placed to cover all myopectineal orifices. In the third group, which comprised patients with direct M3 hernias where the defect was not closed, an XL-sized prosthesis (measuring 12.2 cm × 17.0 cm) was placed to ensure a more extensive overlap compared to the size of the defect.

Our experience in this type of surgical procedure has, over time and with the advancement of materials used for prostheses, led us to avoid fixing the mesh, achieving good results [12, 13].

### Data collection

We collected data from the hospital's electronic records, which encompassed various parameters, such as age, gender, smoking status, ASA grade, hernia side, size of the direct defect (in centimeters), mesh type, mesh size (in centimeters), any intraoperative complications or adverse events, length of hospital stay, occurrence of post-operative seroma, early recurrence (defined as within 1 year), recurrence at 2 years post-operatively, and the duration of the follow-up period. If any values were missing, they were noted as "unknown."

### Follow-up

All patients received appointments at the outpatient clinics within 1–2 months following their surgery to evaluate any potential recurrence or post-operative complications. The diagnosis of recurrence was established through the objective examination of the patient and, if necessary, when the objective examination yielded negative results, through ultrasound of the relevant inguinal region. After the initial visit, patients were scheduled for subsequent clinic appointments at 1 and 2 years for ongoing monitoring. Any instances of early recurrence or post-operative seroma were documented. Recurrences, as per guidelines, were treated with a repair of recurrent inguinal hernia via anterior approach. The two participating centers are geographically close to each other and have historically collaborated in standardizing the surgical technique of TAPP repair. Consequently, they have a homogeneous approach to structuring follow-up, diagnosing, and treating any complications and recurrences. They adhered to the same follow-up protocol, contacting patients via telephone and scheduling an outpatient visit to assess the presence of any recurrences. No loss to follow-up was recorded in this study.

### Statistical analysis

The study included 110 patients with a total of 158 M3 direct inguinal hernias for analysis. These patients were divided into three groups based on their treatment approach: 42 patients underwent primary closure of defects, 73 did not have primary closure and received a standard-size prosthesis, and 43 did not have primary closure but received an XL size prosthesis.

Patients were selected by 1:1 propensity score matching (PSM), matched using age, gender, ASA score, history of smoke, direct defect dimension, and previous hernia repair as covariates in univariable analysis.

We compared demographic and clinical characteristics among these groups. Categorical data, such as recurrent hernia rates, were compared using Chi-square tests or Fisher’s exact tests, while continuous data were analyzed using one-way ANOVA tests as appropriate. Statistical analyses were conducted using Stata MP/15.1 (StataCorp, Lakeway Dr, USA).

## Results

Retrospective data from a prospective database of 2673 patients with inguinal hernia to our department were analyzed. A total of 765 hernias were corrected through laparoscopic procedures, out of which 158 were classified as M3 according to the EHS classification. Out of the total, nine patients (5.7%) had recurrent direct hernias and all of them had previously undergone open mesh repair during their initial hernia surgery. Patients were divided into 3 groups: 24 (21.81%) underwent primary closure of the direct defect before mesh placement, while 54 patients (49.09%) did not undergo primary closure and received a standard-size mesh. 32 patients (29.09%) did not undergo primary closure and were fitted with an XL size mesh.

The average age of the patients was 62 years, ranging from 41 to 84 years, and the majority were male (91.8%). When comparing the three patient groups, no significant differences were noted in terms of demographics, mean operative time, or ASA grade (see Table 1). Univariable analysis, when comparing the three groups, identified significant imbalances in age (*p* = 0.006), gender (*p* = 0.043), ASA score (*p* = 0.006), history of smoke (*p* = 0.007), direct defect dimension (*p* = 0.001), and previous hernia repair (*p* = 0.042). To try to mitigate the impact of possible confounding bias, we proceeded to use propensity score matching at a 1:1 ratio. Eventually 22 patients for each group were analyzed: 22 patients (40 hernias) underwent closure of the defect; 22 patients (39 hernias) did not undergo closure and used a standard-sized mesh; 22 patients (27 hernias) did not undergo closure and used a XL-sized mesh (Table 2).

**Table 1:** Patient demographics and hernia characteristics between groups

	Closure (standard prosthesis) 24 patients, 42 herniae	Non-closure (standard prosthesis) 54 patients, 73 herniae	Non-closure (XL prosthesis) 32 patients, 43 herniae	*p* value
Mean age, year (SD)	62.6 (6.7)	59.4 (8.2)	63.8 (5.6)	0.173
Gender, *n* (%)				
Female	3 (12.5)	5 (9.2)	1 (3.12)	
Male	21 (87.5)	49 (90.8)	31 (96.88)	0.413
Smoking, *n* (%)				
No	9 (37.5)	15 (27.7)	11 (34.37)	
Yes/Ex-smoker	15 (62.5)	39 (72.2)	21 (65.62)	0.65
ASA grade, *n* (%)				
1 + 2	22 (91.6)	48 (88.9)	30 (93.7)	
3	2 (8.3)	6 (11.1)	2 (6.2)	0.742
Previous hernia repair, *n* (%)				
No	40 (95.23)	70 (95.89)	39 (90.69)	
Yes	2 (4.76)	3 (4.1)	4 (9.3)	0.484
Direct hernia defect size (cm)	3.2 (0.8)	3.1 (0.9)	3.0 (0.6)	0.487

**Table 2:** Propensity score

	Closure (standard prosthesis) 22 patients, 40 herniae	Non-closure (standard prosthesis) 22 patients, 39 herniae	Non-closure (XL prosthesis) 22 patients, 27 herniae	*p* value
Mean age, year (SD)	62.3 (6.7)	61.4 (8.2)	62.8 (5.6)	0.573
Gender, *n* (%)				
Female	2 (9.1)	3 (13.6)	5 (22.7)	
Male	20 (90.9)	19 (86.4)	17 (77.3)	0.438
Smoking, *n* (%)				
No	7 (31.8)	3 (13.6)	5 (22.7)	
Yes/Ex-smoker	15 (68.2)	19 (86.4)	17 (77.3)	0.355
ASA grade, *n* (%)				
1 + 2	20 (91.6)	18 (88.9)	19 (93.7)	
3	2 (8.3)	4 (11.1)	3 (6.2)	0.679
Previous hernia repair, *n* (%)				
No	20 (90.9)	19 (86.4)	16 (72.7)	
Yes	2 (9.1)	3 (13.6)	6 (27.3)	0.242
Direct hernia defect size (cm)	3.3 (0.5)	3.1 (0.7)	3.2 (0.3)	0.687

No intraoperative complications occurred. The average operation time was 58 min (SD = 18.7 min) in the closure group, 53 min (SD = 19.3 min) in the non-closure group with standard-size prosthesis, and 55 min in the non-closure group with the XL prosthesis (SD = 21.1). Direct hernia defect sizes were similar between groups (3.3 cm vs. 3.1 cm vs. 3.2, *p* = 0.487). A standard-size prosthesis (10.3 cm × 15.7 cm polypropylene, large pore, 3DMaxTM Mesh, BD, 100 Crossings BoulevardWarwick, Rhode Island 02886, United States) was used in the first and second groups, while the same XL-sized (12.2 cm × 17.0 cm) prosthesis was employed in the third group.

In the 1-year following the operation, there were a total of 5 recurrences of direct (medial) hernias: all of these occurred in the non-closure group with standard-sized mesh (5/66 hernias, 7.5% recurrence rate).

In contrast, there were no recurrences observed in the closure group and in the XL-meh group: this difference was statistically significant (*p* = 0.044). Further paired chi-squared tests were conducted to compare the various techniques: a statistically significant difference was observed between the closed group and the non-closed group with standard-sized mesh (*p* = 0.017) and between the XL-mesh group and the non-closed group with standard-sized mesh (*p* = 0.017).

At 2 years, two additional recurrences were observed: the first one in the group that had placed a standard-sized prosthesis and had not closed the hernia defect (6/66 hernias, 9.09% recurrence rate) and the second one in the non-closure XL-mesh group (1/66 hernias, 1.51% recurrence rate) (Table 3). The results at 2 years confirm what has already been asserted when comparing the three techniques (*p* = 0.07). Further paired chi-squared tests were conducted to compare the various techniques: a statistically significant difference was observed between the closed group and the non-closed group with standard-sized mesh (*p* = 0.008) and between the non-closed XL-mesh group and the non-closed group with standard-sized mesh (*p* = 0.039); a non-statistically significant difference was observed between the closed group and the non-closed group with XL-sized mesh (*p* = 0.311).

**Table 3:** Postoperative and long-term follow-up results between propensity score matched groups

	Closure (standard prosthesis) 22 patients, 40 herniae	Non-closure (standard prosthesis) 22 patients, 39 herniae	Non-closure (XL prosthesis) 22 patients, 27 herniae	*p* value
Length of stay (days)	1 (0.2)	1.1 (0.3)	1.3 (0.6)	0.673
Mean operative time (SD)	58 (18.7)	53 (19.3)	55 (21.1)	0.773
Seroma complication, *n* (%)				
No	21 (95.45)	19 (86.36)	17(77.27)	
Yes	1 (4.54)	3 (13.63)	5 (22.72)	0.213
Recurrence of hernia 1 year, *n* (%)				
No	22 (100)	17(90.41)	22(100)	
Yes	0 (0)	5 (9.58)	0 (0)	0.044
Closure (standard prosthesis) vs. Non-closure (standard prosthesis)	0/22	5/17		0.017
Closure (standard prosthesis) vs. Non-closure (XL prosthesis)	0/22		0/22	
Non-closure (standard prosthesis) vs. Non-closure (XL prosthesis)		5/17	0/22	0.017
Recurrence of hernia 2 years, *n* (%)				
No	22 (100)	16 (72.72)	21 (95.45)	
Yes	0 (0)	6 (27.27)	1 (4.54)	0.007
Closure (standard prosthesis) vs. Non-closure (standard prosthesis)	0/22	6/16		0.008
Closure (standard prosthesis) vs. Non-closure (XL prosthesis)	0/22		1/21	0.311
Non-closure (standard prosthesis) vs. Non-closure (XL prosthesis)		6/16	1/21	0.039

All these cases were subsequently treated with open mesh repair. The average duration until recurrence was 11.2 months.

Regarding seroma formation, there was no significantly higher proportion of patients affected in any of the three groups (4.54% vs. 13.63% vs. 22.72%, *p* = 0.213).

The length of hospital stay was slightly longer in the non-closure group with XL-sized mesh, although this difference was not statistically significant (1.0 vs. 1.1 vs. 1.3 days, *p* = 0.673); the rate of acute post-operative pain was higher in the non-closure group with XL-sized mesh, but not statistically significant (VAS; 1 vs. 1 vs. 4).

Post-operative chronic groin pain at the 1-year mark showed similar rates between both groups (2.3 vs. 2.7 vs. 3.6%).

## Discussion

The objective of this study was to examine the impact of direct defect (M3) closure or XL-sized mesh placement in transabdominal preperitoneal laparoscopic hernioplasty (TAPP) on the occurrence of recurrence and seroma formation. These complications continue to pose significant challenges in hernia surgery, leading to patient discomfort, pain, the potential for incarceration, and the necessity for additional surgical interventions, all of which can be distressing for patients.

These different techniques for managing M3 hernias arise from concerns about inadequate prosthesis overlap, on the one hand, and concerns about an increased risk of complications due to higher tension or potential vascular injuries when closing the hernia defect with a suture, on the other hand. Therefore, over time, in our departments, there was a shift from standard management with normal-sized prostheses and a significant recurrence rate to the placement of larger prostheses that provided greater overlap, resulting in a decrease in recurrence rates. Finally, with the development of advanced laparoscopic skills, the technique was standardized to include suturing of the defect, leading to a low number of recurrences and, most importantly, post-operative complications.

However, there are limited available data concerning the laparoscopic closure of inguinal direct hernia defects. To date, a recent meta-analysis on this topic has been published, encompassing four studies. It is worth noting that only one of these studies was randomized, and it had a maximum follow-up duration of 3 months.

Zhu et al.'s RCT study [14] reported no recurrences in both the control and intervention groups over the 3-month follow-up. However, this short follow-up period may miss late recurrences and has limited validity. Li and Zhang's prospective study [15] found no recurrences in patients who underwent defect closure, but it lacked control groups.

Usmani et al. [16] discovered a significantly lower recurrence risk with closure of medial hernia defects during laparoscopic hernioplasty. This study had the largest sample size and a nine-month follow-up, but it was neither blinded nor randomized. Ng et al. [10] observed a reduction in recurrence risk during the 1-year follow-up. However, this reduction was not statistically significant, primarily due to the small sample size. Our findings align with those computed in the meta-analysis concerning recurrences. There is a statistically significant advantage to defect closure compared to non-closure if a standard dimension prosthesis is used. Possible causes could encompass mesh migration, mesh shrinkage or failure, or insufficient medial overlap during the initial placement. The rationale behind this suturing is to ensure the mesh has a uniformly supportive surface and proper coverage of the myopectineal orifice. For this reason, it is intriguing to assess the utilization of a larger prosthesis. Closing the defect necessitates advanced laparoscopic skills and expertise. Therefore, the option of placing a larger prosthesis to ensure proper overlap can be a highly compelling solution.

Regarding specifically the third group and the mesh size, according to Knook et al. [6], a minimum 3 cm mesh overlap length is advised for orifices larger than 3 cm in diameter. Drawing from the earlier study conducted on pigs, Hiratsuka et al. published an article in which they recommend that hernias with a diameter of 3 cm or more should be repaired using a prosthesis measuring at least 15.6 × 13 cm [7]. Regrettably, our limited sample size hinders us from deriving statistically significant conclusions on the subject, despite the recurrence rate aligning with the first group.

With regards to post-operative seroma formation following laparoscopic inguinal hernia repair, numerous techniques have been documented in the literature with the aim of diminishing the incidence of seroma formation. Our technique is akin to those described by Ng et al., Li et al., and Usmani et al., and our post-operative seroma formation rate (7.5%) aligns with that reported by these authors.

Usmani et al.'s prospective cohort study [16] revealed a noteworthy reduction in post-operative seroma risk during the 1-year follow-up. In contrast, Zhu et al.'s randomized-controlled trial [14] demonstrated a significant decrease in seroma formation at the three-month follow-up. Additionally, the prospective study conducted by Li and Zhang [15] reported just one instance of post-operative seroma among the 36 hernia repairs examined.

On the other hand, Ng et al.'s retrospective cohort study [10] indicated an increase in seroma formation following defect closure. Indeed, this meta-analysis does not identify a statistically significant difference in terms of post-operative seroma risk. In our own study, we observe a similar outcome, the group in which the defect is closed demonstrates the lowest seroma rate. However, this difference does not attain statistical significance, likely attributed to the small sample size.

While the results of this study are promising, they are tempered by its retrospective nature and a relatively small sample size. It is crucial to note that the limited number of surgeons performing the procedure in this study, coupled with the temporal succession of different techniques, may introduce a bias that renders the data susceptible to potential errors.

In addition, there was an initial risk of confounding bias, and therefore, it was decided to use propensity score matching to make this risk lower and acceptable. The use of PSM has been acknowledged as a reliable tool for mitigating selection bias in non-randomized studies and minimizing heterogeneity within study groups when comparing outcomes of interest [17]. A multi-center randomized-controlled trial with a long-term follow-up, encompassing a significant patient cohort and comparing the closure of direct hernia defects with non-closure using both standard-sized and XL mesh, holds the potential to offer substantial insights. Such a comprehensive study could empower us to derive more conclusive and reliable findings, which, in turn, might have implications for the future approach to laparoscopic repairs for direct hernias.

## Conclusions

In the case of M3 hernias, a lack of adequate overlap substantially elevates the risk of recurrence unless the gap is closed by suturing. To achieve optimal overlap, surgeons can utilize their laparoscopic skills to suture the hernia defect. Alternatively, they may choose to position a larger-sized prosthesis, ensuring sufficient coverage relative to the defect's margin.

## Data Availability

Data-sharing requests will be considered by the management group upon written request to the corresponding author. If agreed, deidentified participant data will be available, subject to a data-sharing agreement.
